# Supplementary material for: A Late Cretaceous true polar wander oscillation
Source: Nat Commun. 2021 Jun 15;12:3629. doi: 10.1038/s41467-021-23803-8 (PMC8206135; doi:10.1038/s41467-021-23803-8)
Supplement: Supplementary file 1 — Supplementary Information [file 41467_2021_23803_MOESM1_ESM.pdf]

Supplementary Information for:

A Late Cretaceous true polar wander oscillation

Ross N. Mitchell*, Christopher J. Thissen, David A.D. Evans,

Sarah P. Slotznick, Rodolfo Coccioni, Toshitsugu Yamazaki, and Joseph L. Kirschvink

This file includes:

Supplementary Figures 1-10

Supplementary references

Supplementary Data 1-3 (included as separate spreadsheets)

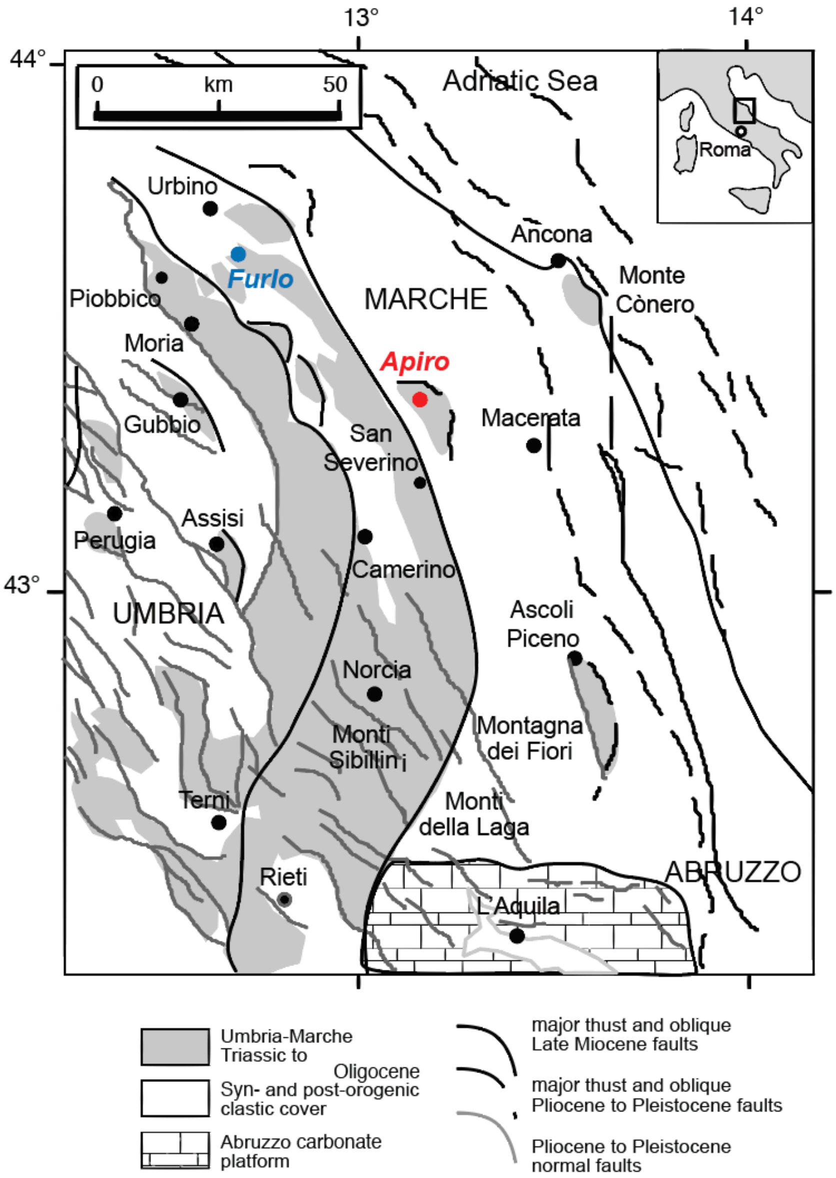

Supplementary Fig. 1 | Geology of sampling localities. Geologic map of sampling sites (Apiro and Furlo sections) within the Late Cretaceous Scaglia Rossa limestone of the Umbria-Marche succession. Modified from Bice et al.[1].

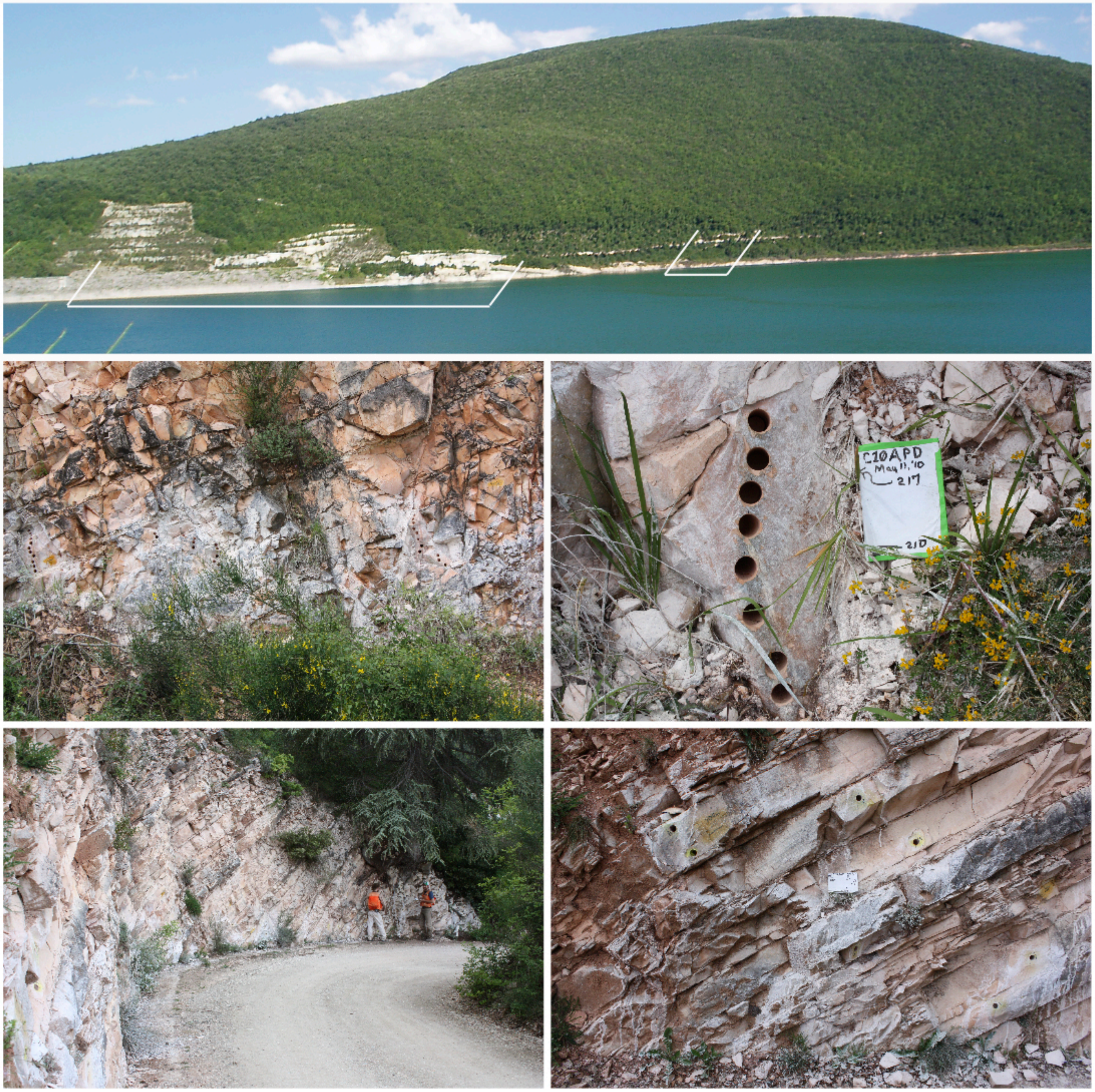

Supplementary Fig. 2 | Outcrops. (Top) Apiro dam road cut section dipping ~26˚ southwest. Sections of exposure are highlighted with white brackets. Note covered interval near base of section that obscures the base of C33r, which is why we supplemented Apiro with our sampling at Furlo. Active tectonics and a high degree of fracturing prevented us from drilling a continuous core to cover this covered interval at Apiro, where an attempt was made across the reservoir, more or less from where this photo was taken. (Middle) High-resolution sampling at Apiro. Note thick beds and lack of pseudo-bedding. Palaeomagnetic samples are ~3 cm in diameter for scale. (Bottom) High-resolution sampling at Furlo, where pseudo-bedding gives the appearance of more regular, smaller-scale bedding than at Apiro. Palaeomagnetic samples are ~3 cm in diameter. In all images stratigraphic bedding dips to the left.

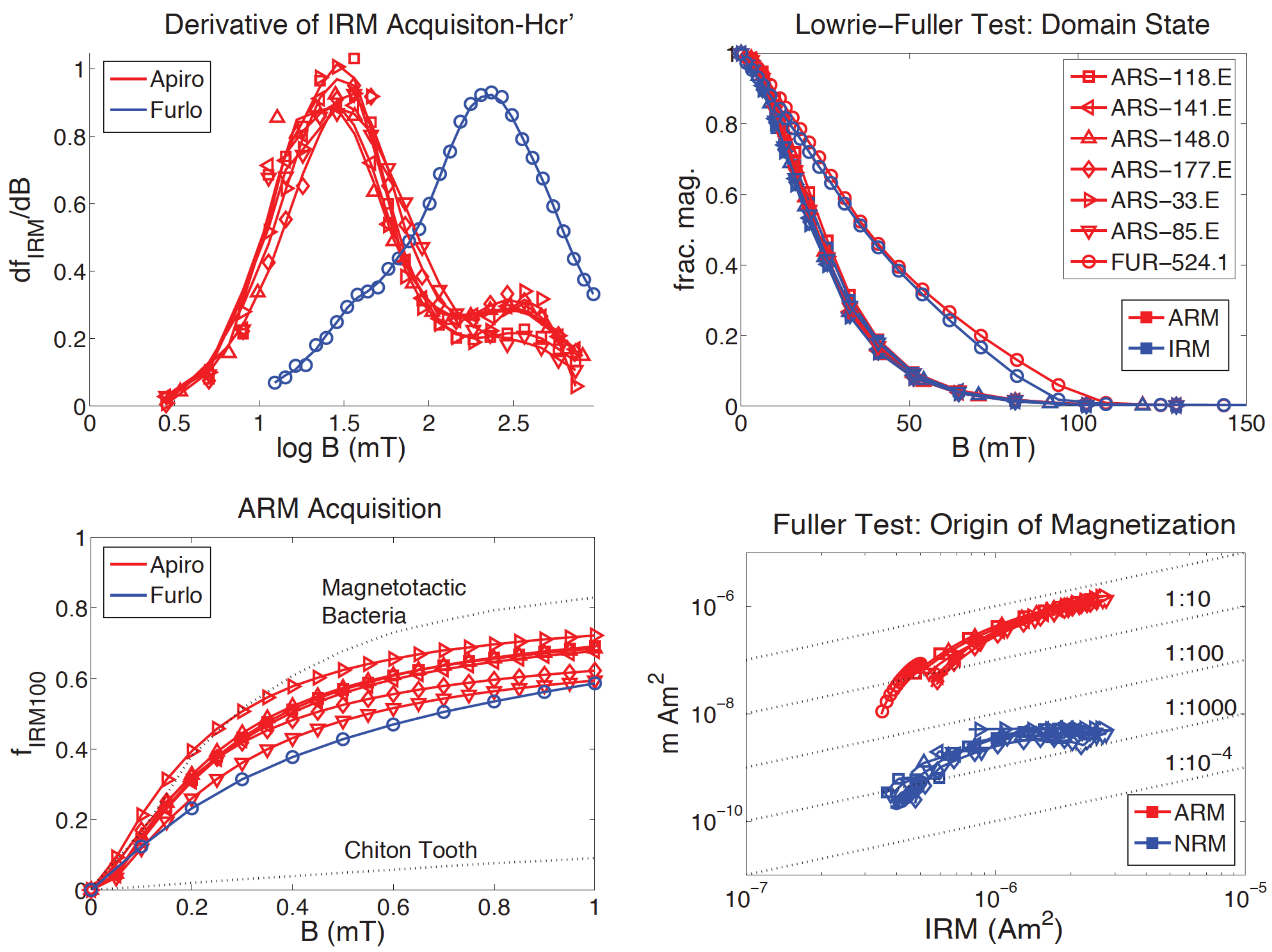

Supplementary Fig. 3 | Rock magnetic experiments by coercivity. 2G SQuID magnetometer rock magnetic experiments on 6 samples from Apiro and 1 sample from Furlo. Color coding key is shown for each plot, and symbols are the same for each sample as listed in the upper right hand corner key. (Top left) Derivative of the IRM acquisition can be used to determine the coercivity spectrum of each sample[2-4]. Here we can see two phases one of mid-coercivity (interpreted to be magnetite) and one of higher coercivity (hematite), as well as their relative proportions within the samples. (Top right) The Lowrie-Fuller Test is a rough estimate for domain state (and thus size and stability) of the magnetic carriers[5,6]. Here the IRM is softer than the ARM for all samples, indicating a single domain-type result. (Bottom left) ARM acquisition can be plotted to understand interaction between grains with the two endmember standards of a non-interacting intact magnetotactic bacteria and a strongly interacting chiton tooth[7]. The Scaglia Rossa samples fall closer to the non-interacting magnetofossil endmember. (Bottom right) The Fuller test is an empirically calibrated test (for magnetite) to understand the origin of magnetization in a sample[8,9]. In sedimentary rocks, weak NRM:IRM ratios of 1:1000 are characteristic for detrital remanent magnetization while higher ratios of 1:10 would signify chemical overprinting.

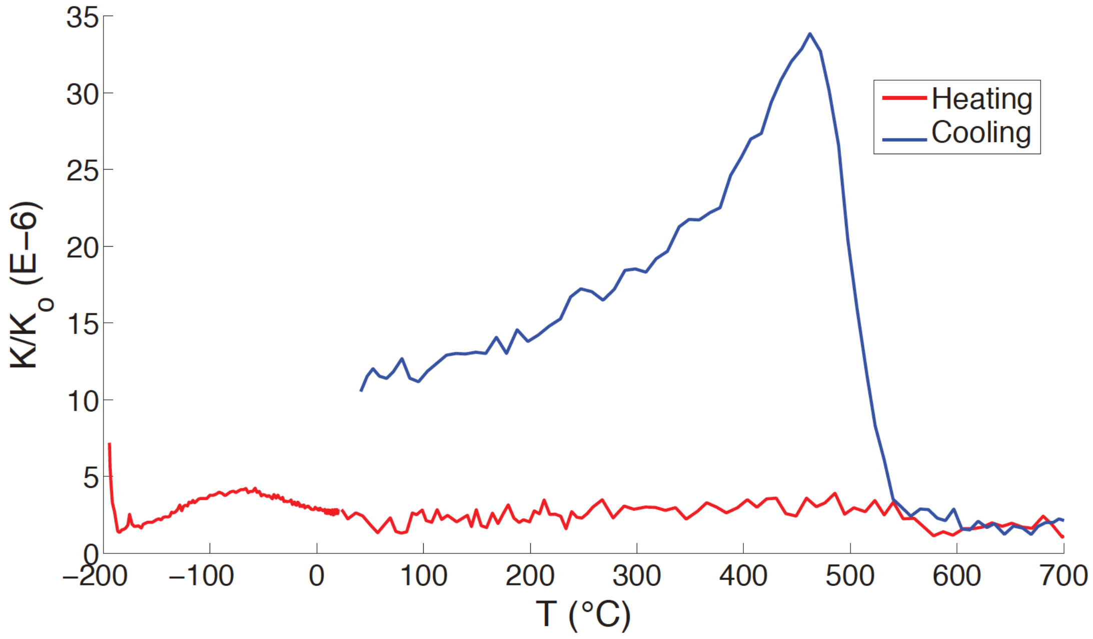

Supplementary Fig. 4 | Rock magnetic experiment by thermal susceptibility. Thermal susceptibility of a sample from the Apiro section (ARS-10.E). Red line shows warming and then heating curve, while blue shows cooling curve.

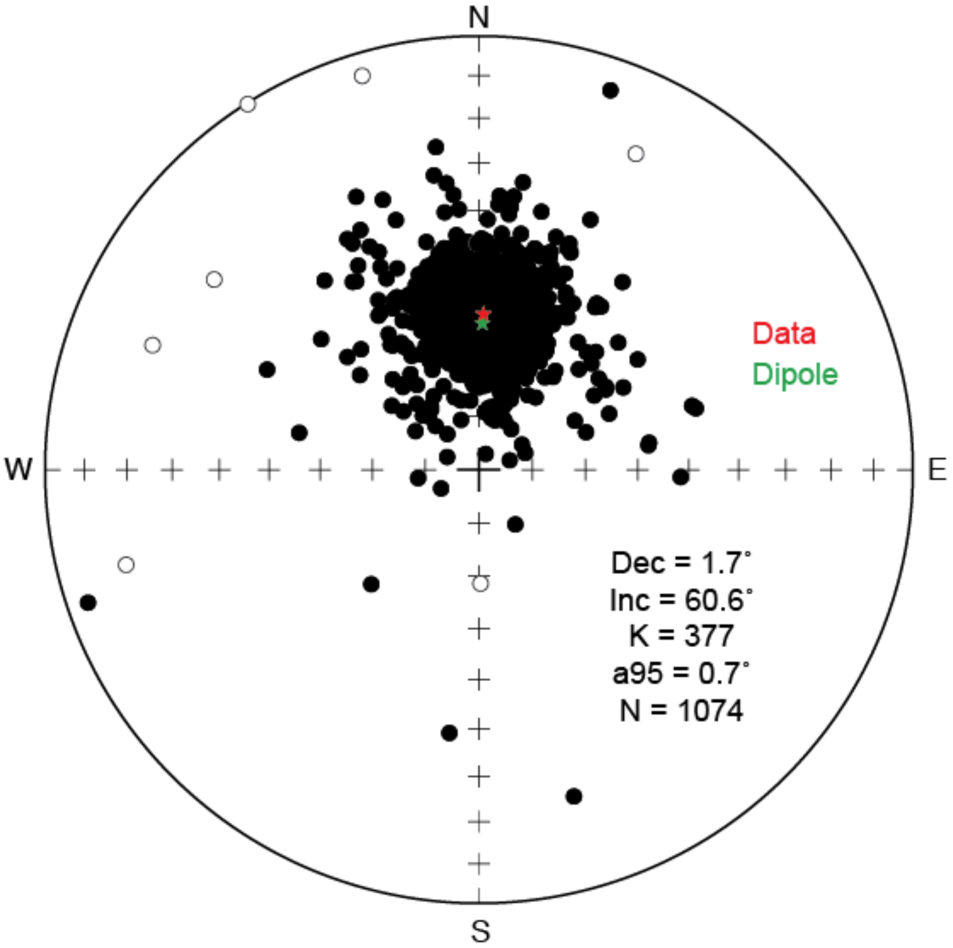

Supplementary Fig. 5 | Palaeomagnetic overprints removed. Equal-area stereonet of low-temperature (up to ~150˚C) palaeomagnetic overprint (Fig. 1a, b) directions from all samples (red star), coincident with the present-local field (green star). Data in Supplementary Data 2.

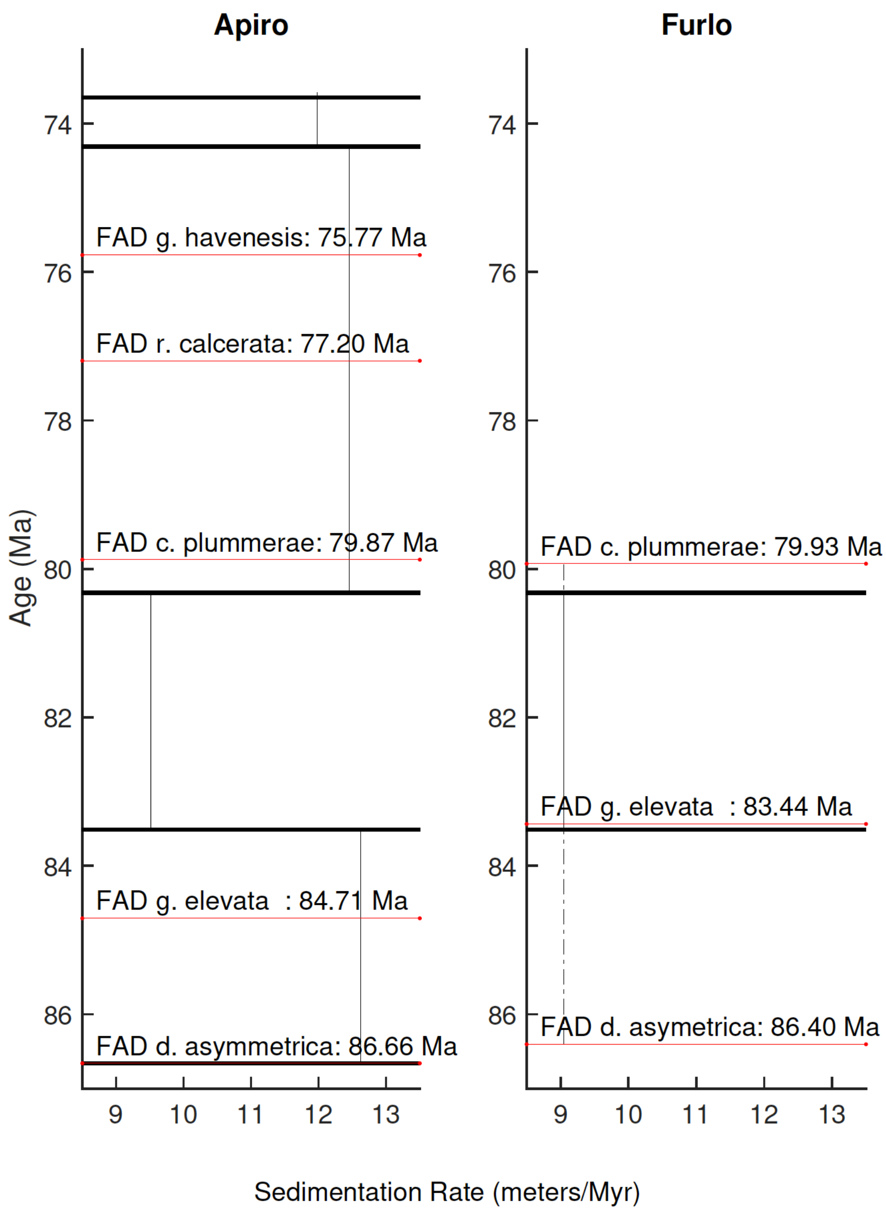

Supplementary Fig. 6 | Age models for Apiro and Furlo sections. Tie points used to generate age models (black horizontal lines; see Methods) with linear interpolation/extrapolation and infer sediment accumulation rates for both sections (black vertical lines measured along horizontal axis). Ages for the *G. elevata* and *D. asymmetrica* zones are constrained two-fold by (1) reversed magnetostratigraphic data attributable to C33r at Apiro that cannot be older than the ca. 83.5 Ma C34n-C33r reversal[10] and (2) the lowest C34n data should not be older than the FAD *D. asymmetrica* that is constrained elsewhere[11]. Both these ages are manually set. Biostratigraphic levels for which new chronostratigraphic ages can be calculated are shown in red, for example the new ages for *C. plummerae*, *R. calcerata*, and *G. havenesis* from Apiro. Inferred sediment accumulation rates are very similar to those estimated for the Upper Cretaceous of the Umbria-Marche basin previously.

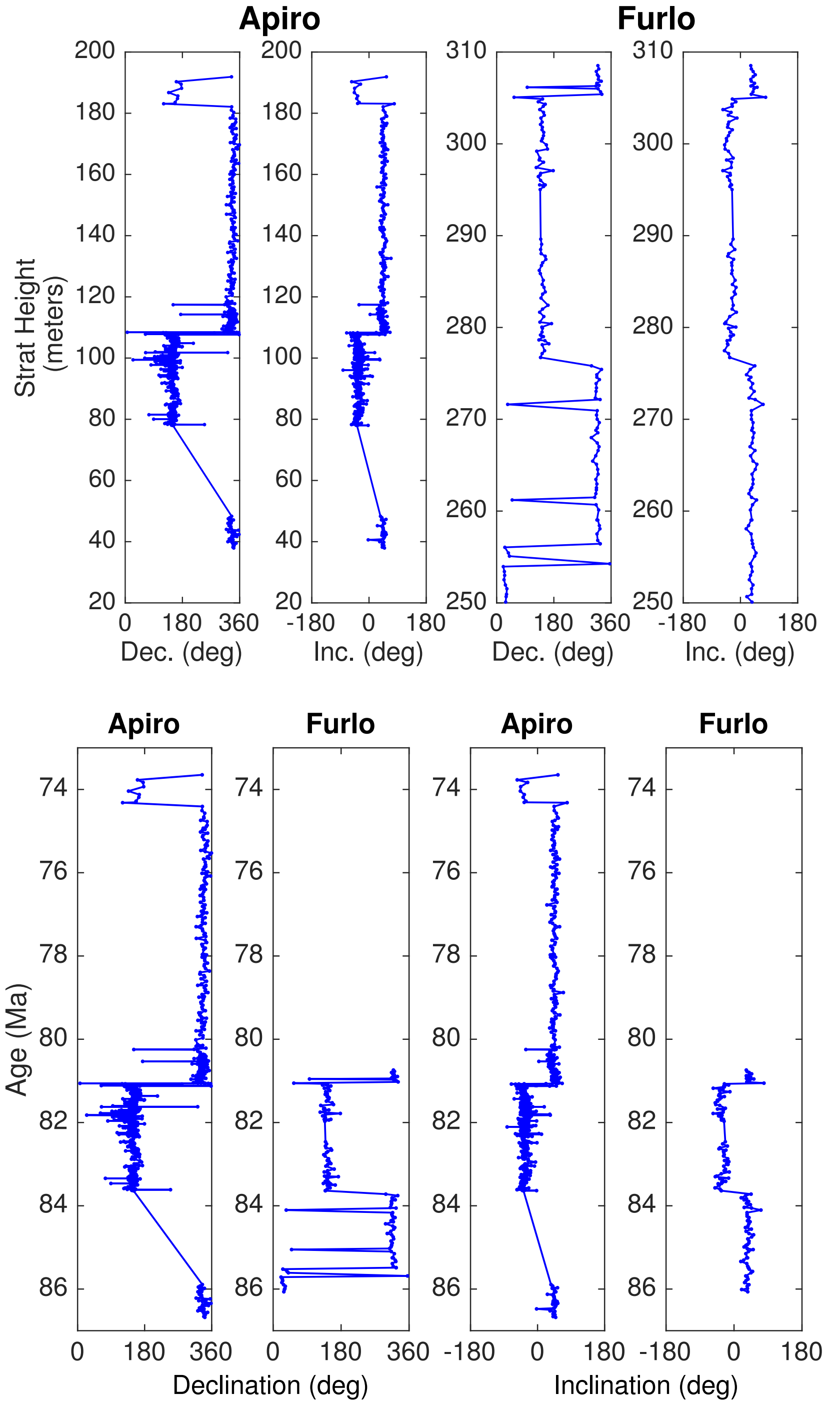

Supplementary Fig. 7 | Raw palaeomagnetic data of Apiro and Furlo sections. Data according to (top) stratigraphic height and (bottom) age as inferred from age constraints in Supplementary Figure 6 (see Fig. 2). Aberrant declinations near 0° in Furlo between 250-275 m are due to slump rotations and were excluded from directional analysis. Data in Supplementary Data 1.

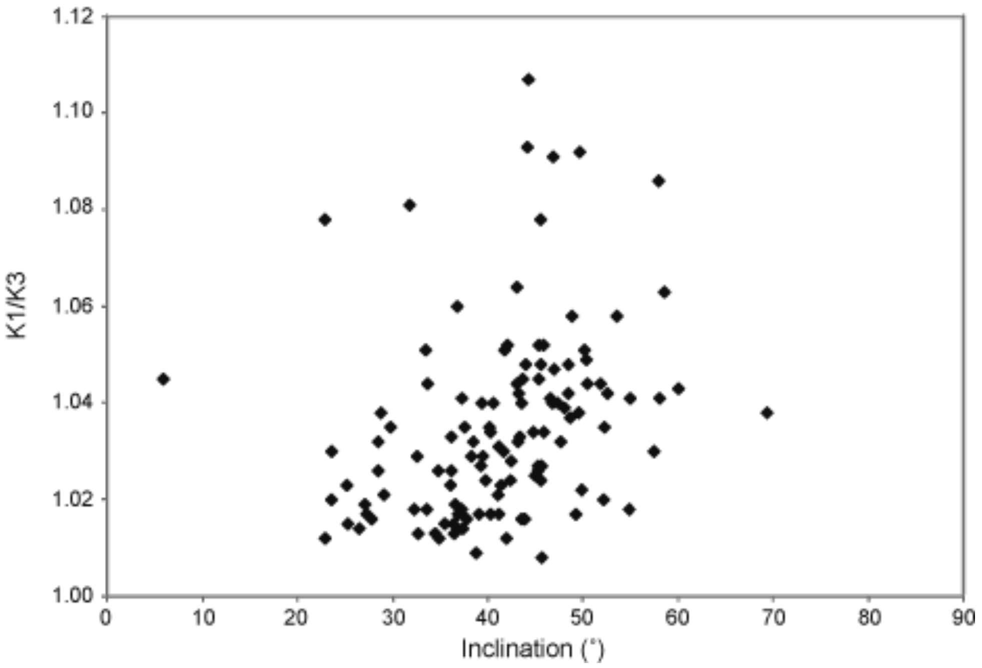

Supplementary Fig. 8 | Cross-plot of palaeomagnetic inclination and anisotropy of magnetic susceptibility (AMS) data. AMS data from the Apiro section plotted against inclination, in order to test whether inclination swings are a function of varying flattening fabric. Larger K1/K3 values equals more anisotropic susceptibility. The correlation is very weak (r^2 = 0.05), confirming that inclination cycles cannot be explained by varying degrees of inclination flattening.

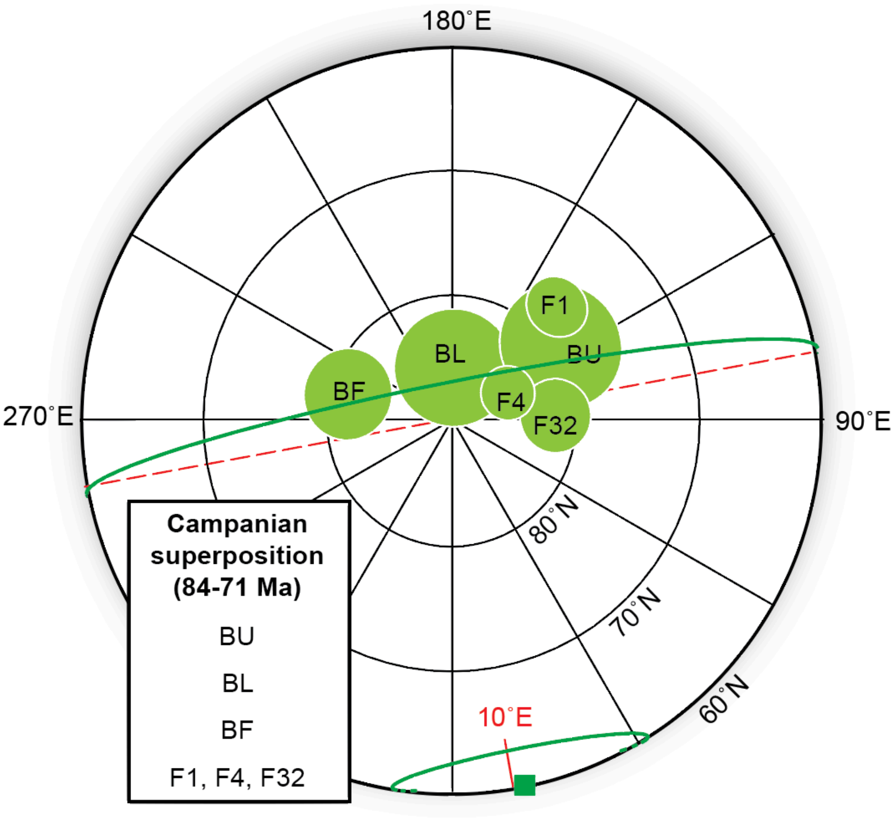

Supplementary Fig. 9 | Palaeomagnetic data at ca. 80 Ma. from Aix-en-Provence in France. Data are from ref. [12]. For tectonic restoration at 80 Ma, the Euler rotation for Europe was applied[13]. Note that the orientation of pole dispersion in the French poles is consistent with that of the Italian poles and modern true polar wander (red; Fig. 3). See figure 2 of Sager and Koppers[14] for summary of existing poles from North America[15-18].

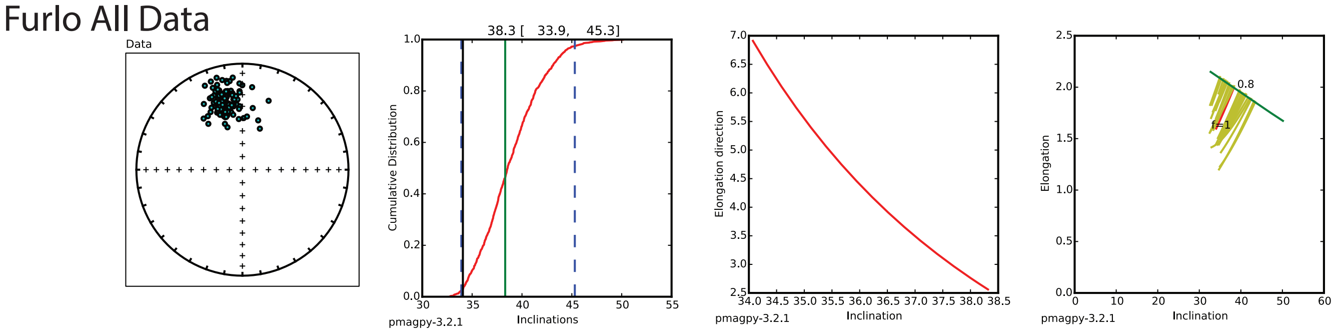

Supplementary Fig. 10 | Elongation/inclination analysis of inclination shallowing at Furlo. A flattening factor of 0.8 is consistent with 0.85 as based on comparison between sections and with the long-term tectonic trend in inclination of north Umbria (Fig. 2).

Supplementary References

1 Bice, D. M., Montanari, A. & Rusciadelli, G. Earthquake-induced turbidites triggered by sea level oscillations in the Upper Cretaceous and Paleocene of Italy. *Terra Nova* **19**, 387-392 (2007).

2 Peters, C. & Dekkers, M. Selected room temperature magnetic parameters as a function of mineralogy, concentration and grain size. *Physics and Chemistry of the Earth* **28**, 659-667 (2003).

3 Heslop, D., Dekkers, M. J., Kruiver, P. P. & Van Oorschot, H. M. Analysis of isothermal remanent magnetization acquistion curves using the expectation-maximization algorithm. *Geophysical Journal International* **148**, 58-64 (2002).

4 Robertson, D. J. & France, D. E. Discrimination of remanence-carrying minerals in mixtures, using isothermal remanent magnetization acquisition curves. *Physics of the Earth and Planetary Interiors* **82**, 223-234 (1994).

5 Lowrie, W. & Fuller, M. On the alternating field demagnetization characteristics of multidomain thermoremanent magnetization in magnetite. *Journal of Geophysical Research* **76**, 6339-6349 (1971).

6 Xu, S. & Dunlop, D. J. Toward a better understanding of the Lowrie - Fuller test. *Journal of Geophysical Research: Solid Earth* **100**, 22533-22542 (1995).

7 Cisowski, S. Interacting vs. non-interacting single domain behavior in natural and synthetic samples. *Physics of the Earth and Planetary Interiors* **26**, 56-62 (1981).

8 Fuller, M., Kidane, T. & Ali, J. AF demagnetization characteristics of NRM, compared with anhysteretic and saturation isothermal remanence: an aid in the interpretation of NRM. *Physics and Chemistry of the Earth* **27**, 1169-1177 (2002).

9 Fuller, M. *et al.* NRM: IRM(S) demagnetization plots; An aid to the interpretation of natual remanent magnetization. *Geophysical Research Letters* **15**, 518-521 (1988).

10 Wu, H. *et al.* Cyclostratigraphy and orbital tuning of the terrestrial upper Santonian-Lower Danian in Songliao Basin, northeastern China. *Earth and Planetary Science Letters* **407**, 82-95 (2014).

11 Gradstein, F. M., Ogg, J. G., Schmitz, M. D. & Ogg, G. *The Geologic Time Scale 2012*. (Elsevier, 2012).

12 Westphal, M. & Durand, J. P. An Upper Cretaceous paleomagnetic poles for stabli Europ from Aix-en-Provence (France). *Earth and Planetary Science Letters* **94**, 143-150 (1989).

13 Torsvik, T. H. *et al.* Phanerozoic polar wander, palaeogeography and dynamics. *Earth-Science Reviews* **114**, 325-368 (2012).

14 Sager, W. W. & Koppers, A. A. P. Late cretaceous polar wander of the Pacific plate: Evidence of a rapid true polar wander event. *Science* **287**, 455-459 (2000).

15 Swenson, P., M. McWilliams. Paleomagnetic results from the Upper Cretaceous Maudlow and Livingston Formations, southwest Montana. *Geophysical Research Letters* **16**, 669-672 (1989).

16 Diehl, J. F. The Elkhorn Mountains revisited: New data for the Late Cretaceous paleomagnetic field of North America. *Journal of Geophysical Research* **96**, 9887-9894 (1991).

17 Gunderson, J. A. & Sheriff, S. D. A new late cretaceous paleomagnetic pole from the Adel Mountains, West Central Montana. *Journal of Geophysical Research* **96(B1)**, 317–326 (1991).

18 Acton, G., R.G.Gordon in *American Geophysical Union, Spring Meeting 2005, abstract #GP13B-11*.
