# Supplementary material for: A Late Cretaceous true polar wander oscillation
Source: Nat Commun. 2021 Jun 15;12:3629. doi: 10.1038/s41467-021-23803-8 (PMC8206135; doi:10.1038/s41467-021-23803-8)
Supplement: Supplementary file 3 — Description of Additional Supplementary Files [file 41467_2021_23803_MOESM3_ESM.docx]

**Description of Additional Supplementary Files**

File name: Supp Data 1 ChRMs.xlsx

Description: Characteristic remanent magnetizations (ChRMs) of the Scaglia Rossa Limestone.

File name: Supp Data 2 PLFs.xlsx

Description: Present local field (PLF) overprints of the Scaglia Rossa Limestone.

File name: Supp Data 3 poles.xlsx

Description: Palaeomagnetic poles generated and used in this study.
